# Waist-to-height ratio is the best anthropometric predictor of hypertension: A population-based study with women from a state of northeast of Brazil

**DOI:** 10.1097/MD.0000000000005874

**Published:** 2017-01-13

**Authors:** Tainá C.S. Caminha, Haroldo S. Ferreira, Narithania S. Costa, Ricardo P. Nakano, Renata Elyonara S. Carvalho, Antônio F.S. Xavier, Monica L. Assunção

**Affiliations:** a Faculty of Nutrition; b Institute of Biological and Health Sciences, Federal University of Alagoas, Campus A. C. Simões, Cidade Universitária, Maceió, AL, Brazil.

**Keywords:** body mass index, cardiovascular disease, obesity, risk factors, sensitivity, specificity

## Abstract

The WHO recommends the use of some anthropometric parameters as a screening resource for individuals under cardiometabolic risk. However, in the validation of these indicators, Brazilian women were not included. These women have different anthropometric profile compared to women who integrated the samples of the validation studies. We aimed to verify the accuracy of anthropometric indicators as a resource for the screening of women with hypertension. A cross-sectional study, with a probability sample of 3143 women (20–49 years) from the state of Alagoas (northeast of Brazil), was carried out. Hypertension was identified by systolic blood pressure (SBP) ≥140 mm Hg and/or diastolic BP ≥90 mm Hg and/or regular use of antihypertensive drugs. The anthropometric indicators analyzed were BMI, waist circumference, waist-to-hip ratio, waist-to-height ratio (WHtR), body fat percentage, and conicity index. The accuracy definition of the indicators and the identification of best cut-off points were carried out on the basis of ROC curve analysis and Youden index, respectively. The prevalence of hypertension was 21.8%. All indicators used in hypertension identification had area under the ROC curve (AUC) >0.5. The WHtR with cut-off point of 0.54 was the best performance indicator (AUC = 0.72; *P* < 0.05; sensitivity = 67%, specificity = 66%). The WHtR with cut-off point of 0.54 has constituted the most accurate indicator in the screening of women with hypertension. In the absence of specific studies and considering the largest ethnic proximity and environmental/epidemiological similarity, the findings now obtained can be extended to women of other Brazilian states, especially those in the Northeastern region.

## 1 Introduction

Systemic arterial hypertension is one of the most important risk factors for the development of cardiovascular disease (CVD), the main cause of death worldwide.^[1]^ In Brazil, it is estimated that hypertension affects 21.4% of the population. Among women, about 24.2% are affected by the problem.^[2]^

The diagnosis of hypertension is a gradual process that requires a great deal of care during pressure check procedure and demands multiple assessments, at different points in time.^[3]^ Late diagnosis can extend the harmful effects of high blood pressure (BP) on the cardiovascular system, as well as promote the onset of irreversible sequelae.^[4]^

Considering the important participation of obesity in the hypertension etiology, especially when characterized by fat accumulation in the abdominal region,^[5]^ the use of anthropometric indicators related to body adiposity can assist in the screening of individuals at greater risk of hypertension. The individuals thus identified would be referred to better structured health services, enabling the monitoring and the early diagnosis of hypertension and, consequently, optimizing the opportunities in the context of secondary prevention measures.^[6]^

Recognizing the importance of this strategy, some international institutions have recommended the use of anthropometric parameters to identify obesity and associated health risks. The World Health Organization (WHO) has proposed the body mass index (BMI) to identify the overweight, as well as the waist circumference (WC) or waist-to-hip ratio (WHR) to detect the risk of metabolic complications.^[6,7]^ It is important to emphasize that the construction of indicators based on these measures has occurred from data obtained in predominantly Caucasian samples, especially from developed countries.^[7]^

However, the body characteristics from different populations vary, not only according to the genetic load, but also to the environment in which the individual is inserted.^[8]^ Thus, individuals from different ethnicities have different anthropometric characteristics.^[9]^ In order to deal with this situation and to improve the accuracy of anthropometric indicators of abdominal obesity, the International Diabetes Federation established WC cut-offs values according to different ethnicities and geographical regions. However, Brazil was not included in this definition.^[10]^

Due to differences related to body composition and to predisposition to diseases among populations, it is necessary to validate the hypertension anthropometric predictors and also to establish specific cut-off points that optimize the accuracy of these indicators.^[6,7]^ In this respect, few studies have been carried out in Brazil, none of them being conducted in the state of Alagoas.

Considering the importance of hypertension early detection, in order to establish timely care and CVD control, this study aimed to investigate the accuracy of anthropometric indicators as a resource for screening women with high BP.

## 2 Methods

### 2.1 Types of study and sampling plan

This is a cross-sectional study, with a representative probability sample of women aged between 20 and 49, from the state of Alagoas. For this purpose, the hypertension, whose prevalence for Brazilian women was estimated at 24.2%,^[2]^ was the dependent variable in the sample size calculation. The other considered parameters were: sampling error of 2.5%, correction of the complex design effect of 2.0 and an estimated population of 718,493 women.^[11]^ For a 95% confidence interval, it would be necessary a total of 2815 women. To cover any sampling losses, there has been an addition of 10%. Thus, the planned sampling amounted 3097 women. The calculations were carried out in StatCalc module of Epi-info software, version 7.1.4.

A sampling process in multiple stages has been adopted, with 4 steps. In the first step, 20 municipalities, each of them with probability proportional to its population size, were randomly selected. As a second step, 4 census sectors were selected within each municipality, respecting the proportion between urban and rural sectors. Due to its larger population, Arapiraca contributed with 8 sectors and Maceió, the state capital, with 40 sectors. In the third step, one block in each census sector, was randomly selected and, finally, a starting point was also randomly selected within each block, from which 27 consecutive households were visited.

For this study we selected women aged 20 to 49 years. In each household, only one resident should be investigated. When there was more than one woman that met the inclusion criteria, the choice was made randomly.

Therefore, it would be possible to identify a maximum number of 3240 women eligible for the study, considering these criteria and procedures.

### 2.2 Data collection

The data collection was conducted from January to September 2014, through home visits, using structured questionnaires and regularly calibrated equipments, all previously tested in a pilot study. The field team was composed by a general coordinator, a supervisor, 2 anthropometrists and 10 interviewers, all of them properly trained to perform their activities.

During the interviews, socioeconomic and demographic data, and some characteristics such as smoking habits, drink alcoholic beverages, current use of medicines, and salt intake, were investigated.

For the economic classification, the criteria proposed by ABEP^[12]^ were used, which classifies individuals into classes A1, A2, B1, B2, C1, C2, D, and E according to the total score they are given, with A representing the highest class. The total score is the sum of the scores assigned for the number of durable goods in the household, the level of education of the head of household, the number of toilets in the household, the access to public services (piped water and pavement), and the number of domestic workers (maids). It was decided not to use the subclasses, so the women's families were classified into 5 economic groups: A, B, C, D, and E.

The level of physical activity was classified using the International Physical Activity Questionnaire—IPAQ, short version.^[13]^

The BP check was carried out in duplicate, with the individual seated and, after 15 minutes of rest, respecting a minimum interval of 5 minutes between checks. The digital devices used were an Omron brand product, model HEM-7113. In case of having a difference above 5 mm Hg between the 2 measurements, a third check was carried out. In this case, for average calculation, the most discrepant measurement was disregarded.

Hypertension was defined when the mean systolic blood pressure (SBP) was ≥140 mm Hg and/or mean diastolic blood pressure (DBP) was ≥90 mm Hg and/or when the participant stated to be using antihypertensive drugs. The classification of hypertension levels was considered as follows: stage I (SBP between 140 and 159 or DBP between 90 and 99) and stage II (SBP > 160 or DBP > 100).^[3]^ In this categorization, women who reported to be using drugs to control BP were excluded.

The anthropometric data were collected respecting international standardized protocols.^[7]^ A portable digital scale (MS6121R model Charder), with capacity of 250 kg and precision of 100 g, was used to measure body weight. The stature was verified in a stadiometer (model 213, Seca) that had an inextensible tape measure, with sensitivity of 0.1 cm and range of 205 cm. The percentage of body fat (% BF) was estimated by electrical bioimpedance, obtained in bipolar devices, from Omron brand, HBF-306 model.

The circumference measurements were checked using an inextensible tape measure with a sensitivity of 0.1 cm and a capacity of 150 cm. The WC was measured at the midpoint between the last rib and the upper edge of the iliac crest, while the hip circumference was obtained at the point of greater magnitude in this region.

From the values of these measures, the WHR has been calculated, by dividing the waist measurement by the hip circumference^[6]^ and the waist-to-height ratio (WHtR) has been calculated, by dividing the WC by the individuals stature.^[14]^

The conicity index (C index) was calculated by using the following formula^[15]^: 
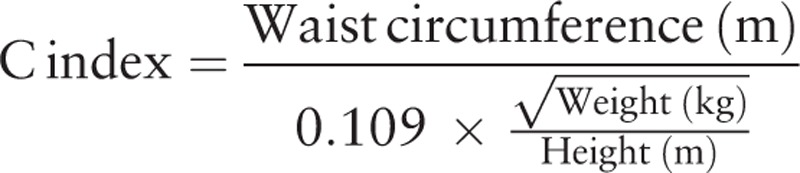


In order to classify the nutritional status, the BMI has been used, establishing the following categories: overweight (from BMI ≥25 to <30 kg/m^2^) and obesity (BMI ≥30 kg/m^2^). Weight excess is the term used to refer to the sum of the cases of overweight and obesity.^[7]^ The cut-off point used in the definition of abdominal obesity, based on WC, was >80 cm.^[6]^

### 2.3 Statistical analysis

The data were entered in separate double entry, in a form generated by the Epi-Info software, version 3.5.4 (CDC, Atlanta, GA, USA). The database was examined in order to exclude outliers (extreme and improbable values) and the statistical analysis was performed with Stata software, version 13.0.

A descriptive analysis for socioeconomic and anthropometric variables and for BP levels was carried out. Correlations between the variables BMI, % BF, C index, WC, WHR, and WHtR, and the levels of SBP and DBP were checked, using the Pearson correlation test. For this test, those participants who stated the use of antihypertensive drugs were excluded, due to these drugs influence on BP levels.

The analysis of ROC curve (receiver operating characteristic) was used to assess the accuracy of the anthropometric indicators, as hypertension predictors. Initially, the areas under the curve (AUC) were calculated and compared for each of the indicators, by using the DeLong test. Subsequently, the sensitivity and specificity values for cut-off points, obtained in the ROC curve analysis, were investigated. From these results, the Youden index (J) was calculated, using the following formula^[16]^: 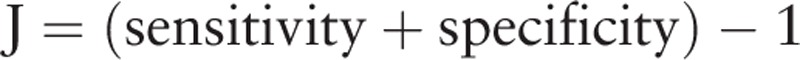


It was assumed that the best cut-off point was the one that obtained the highest value for the Youden index, that is, the one that obtained the highest value for the sum of sensitivity and specificity. The cut-off point thus established will be mentioned in this paper as “the ideal cut-off point,” to distinguish this point to those cut-off points traditionally used.

The associations between high BP and general and abdominal obesity, that were identified respectively from BMI ≥30 kg/m^2^ and WC >80 cm, were analyzed using the Chi-square test and the prevalence ratio (PR), calculated using Poisson regression with robust adjustment of variance.

These same procedures were used to analyze the association between hypertension and the anthropometric parameters, classified according to the cut-off points identified by this study. In addition to the unadjusted analysis, the associations were tested by multivariable analysis, following the hypertension conceptual model, via 2 hierarchical levels, to control possible confounding factors. The first level considered the socioeconomic and demographic variables (age, race, economic class, and education). The second level included variables related to lifestyle (physical activity level, smoking, alcohol consumption, and salt intake).^[17]^ The variables that integrated the second model were adjusted by the variables that remained significant (*P* < 0.05) in the first model. For each level, the adjustment was carried out using the backward process.

The significance level considered for all statistical procedures was of 5% (*P* < 0.05).

### 2.4 Ethical aspects

This study is part of the II Health Diagnosis of Maternal and Child Population of Alagoas State, that was approved by the Ethics Committee on Human Research of the Federal University of Alagoas (case no. 010102/0355).

All surveyed women were informed about the study objectives, its risks and benefits, and expressed their agreement to participate by signing an informed consent form.

## 3 Results

### 3.1 Characteristics of study sample

**Figure 1:**
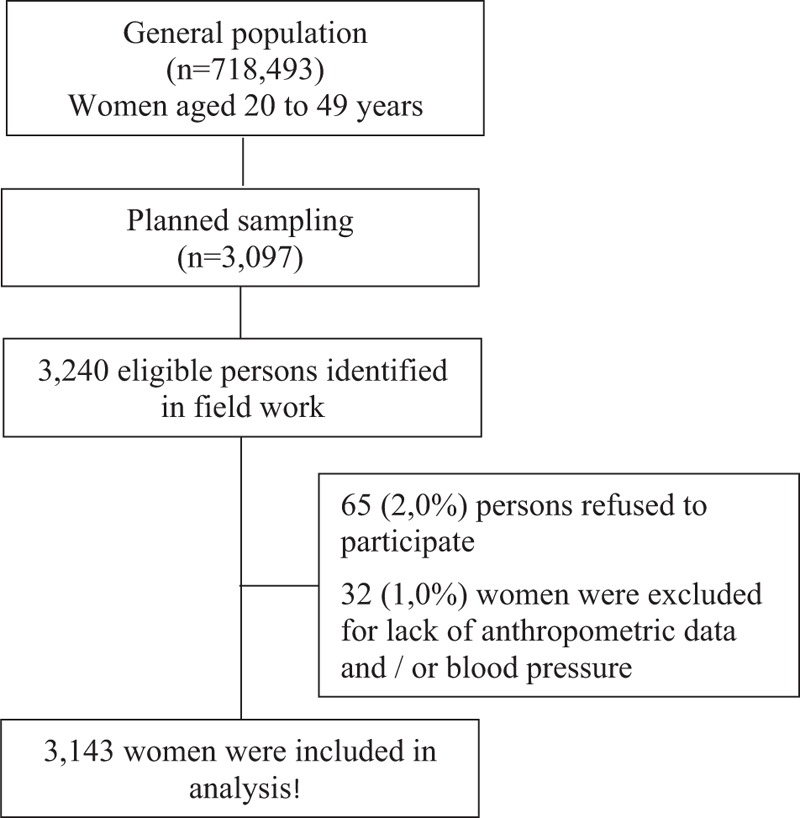
Flow diagram of the participant selection process.

The sample consisted of 3143 women (Fig. 1), of which 75.2% declared themselves as black or dark-skinned. About 73% of them had 5 or more years of education. Among the households visited, 41.8% of them were located in the state capital and 56.2% of women belonged to economic classes D or E. Other demographic and anthropometric characteristics as well as the BP levels of the participants are shown in Table 1.

**Table 1:**
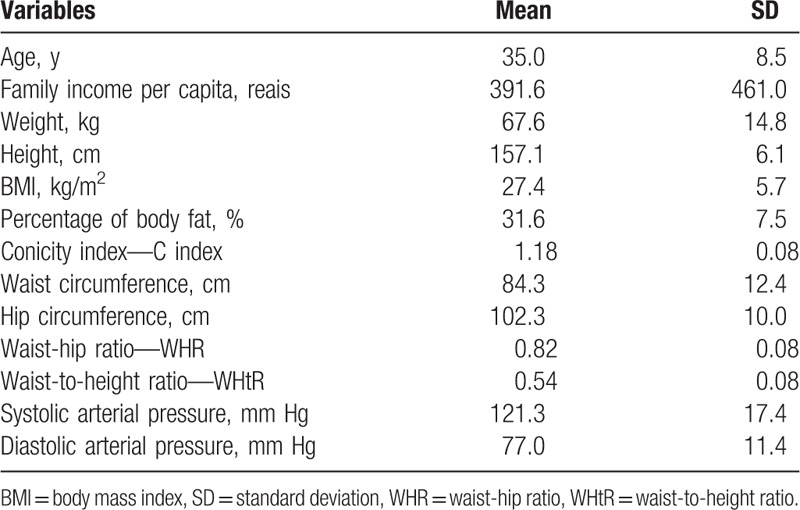
Demographic and anthropometric characteristics and blood pressure levels of women from the state of Alagoas, 2014 (n = 3.143).

The BMI classification revealed that 62.4% of women had weight excess, of which, 28.2% had obesity. The abdominal obesity rate reached 59.2%.

The prevalence of high BP reached 21.8%; from this group, 9.5% refereed to stage I and 3.2% to stage II. The occurrence of this condition was higher among obese women, in comparison to those with normal weight (38.3% vs 15.4%; PR = 2.5; 95% CI: 2.2–2.8). The same occurred in relation to those women who had abdominal obesity (30.4% vs 9.5%; RP = 3.2; 95% CI: 2.7–3.8).

### 3.2 Comparison of measured BP values and anthropometric parameters

There was a statistically significant and positive correlation (*P* < 0.001) among all evaluated anthropometric parameters, and SBP and DBP variables (Table 2). It is worth highlighting that the WHtR and WC parameters presented the highest correlation force.

**Table 2:**
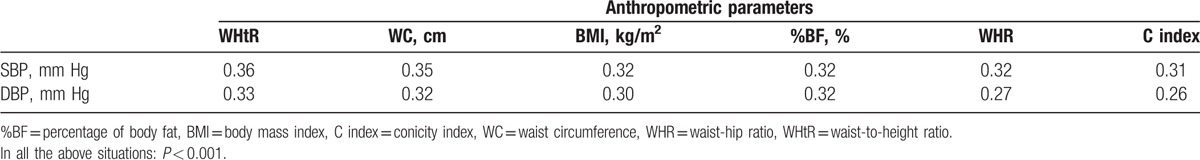
Correlation between systolic blood pressure (SBP) and diastolic blood pressure (DBP), and the anthropometric indicators in women from state of Alagoas, 2014.

### 3.3 Diagnostic accuracy of high blood pressure based on anthropometric indicators

**Figure 2:**
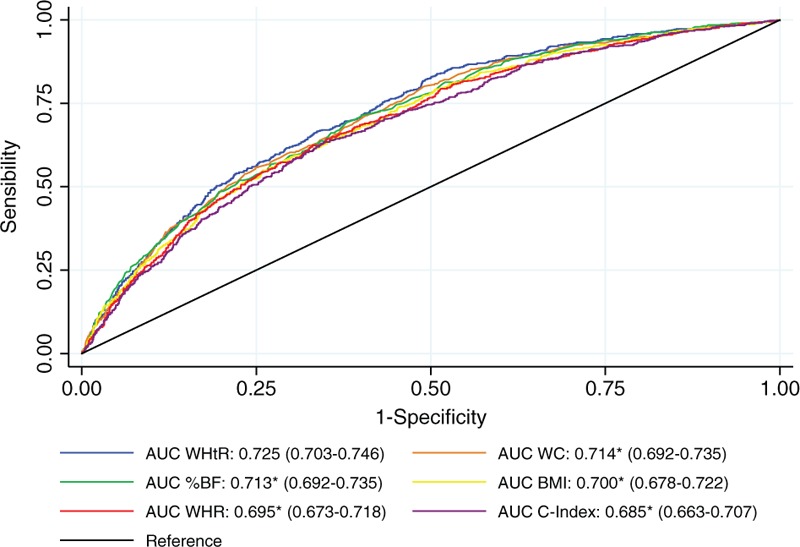
ROC curves for high blood pressure screening in women, through anthropometric indicators, and their respective areas under the curve (95% CI), Alagoas, Brazil, 2014. % BF = percentage of body fat, 95% CI = 95% confidence interval, AUC = area under the curve, BMI = body mass index, C index = conicity index, ROC = receiver operating characteristic, WC = waist circumference, WHR = waist-hip ratio, WHtR = waist-to-height ratio. ∗Significant difference (*P* < 0.05) when compared to AUC (WHtR).

Figure 2 shows the areas under the ROC curve for the performance of each indicator, in relation to the ability to identify high BP. All indicators presented satisfactory performance (AUC > 0.5), and WHtR indicator was the one with greater discriminatory power (AUC = 0.725, *P* < 0.05).

The cut-off points, that were considered as ideal points for optimizing the indicators accuracy, are shown in Table 3; these, in this context, presented better accuracy than those traditionally proposed.

**Table 3:**
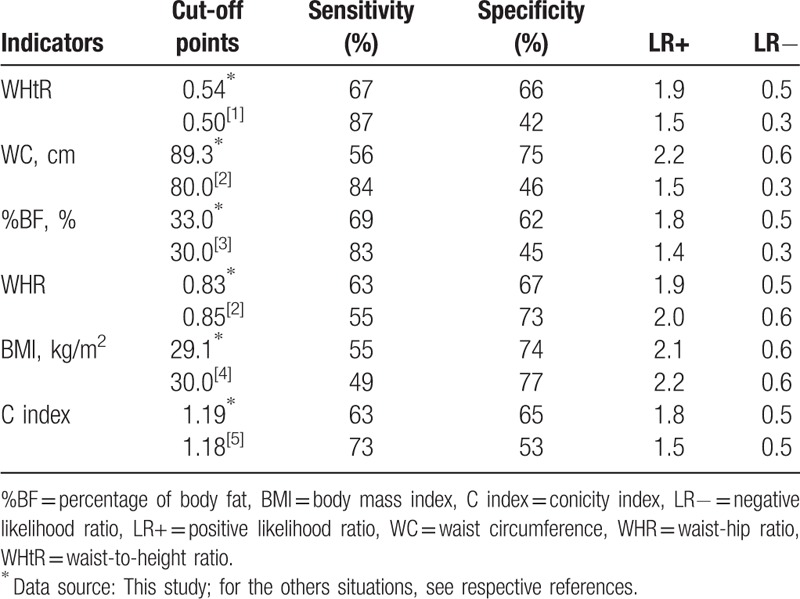
Characterization of the accuracy of anthropometric parameters, according to different cut-off points, as a criterion for identifying high blood pressure in women from state of Alagoas, Brazil, 2014.

Based on the cut-offs points shown in Table 3, the indicators that showed greater sensitivity in high BP identification were WHtR and %BF. On the other hand, the WC and BMI indicators had the highest specificity. The best combination of sensitivity and specificity was observed in the WHtR indicator.

### 3.4 Comparison between cut-off points

The values shown in Table 4 reveal the association between high BP and the anthropometric parameters, categorized according to the cut-off points established as being of better accuracy in this study. In the unadjusted analysis, the risk of high BP was approximately 3 times higher in women with high measures. After the control of possible confounding factors through multivariable analysis, the strength of associations decreased for all parameters, but remained significant in all situations (*P* < 0.001).

**Table 4:**
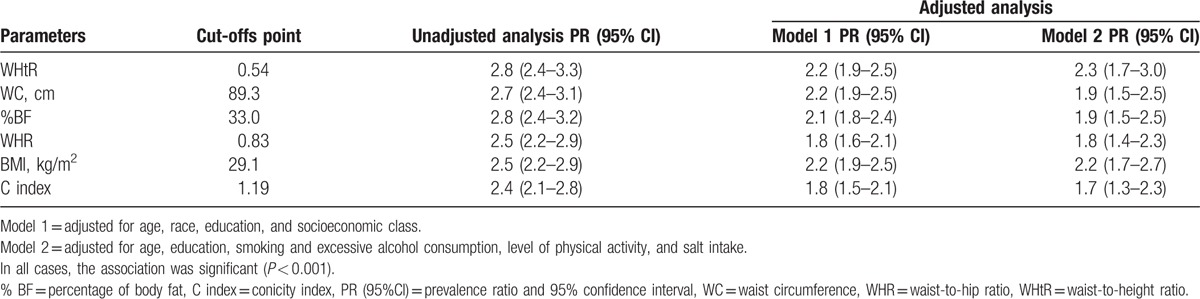
Association between high blood pressure and anthropometric parameters, using specific cut-offs points for women from the state of Alagoas, 2014.

In accordance with the analysis of ROC curves, the WHtR indicator stood out as the best anthropometric indicator to predict high BP, as it showed greater strength of association in all models considered in this study.

## 4 Discussion

Alagoas is a state in the Northeastern region of Brazil characterized by having the worst social indicators, such as the lowest Human Development Index (HDI) in the country.^[18]^ Consistent with this position, this study observed that average per capita income close to half minimum wage and that only 9.0% of women belonged to socioeconomic classes A and B. In the Brazilian population, it is estimated that 25.2% belong to these economic classes^[12]^ and the average per capita income corresponded to 1.4 minimum wages in 2015.^[19]^

These precarious economic conditions are associated with growth deficits.^[8,9]^ Women from Alagoas presented body height around 3 cm below the national average^[11]^ and 5 cm below the US average.^[20]^ The body height in adulthood is the result of a complex interaction between genes and environment. In unfavorable conditions, such as those experienced by a large part of the population from Alagoas, the linear growth does not reach all its genetic potential because, in most cases, this group did not have their nutritional requirements fully met at crucial moments of their lives.^[8]^

All these conditions determine differences in anthropometric profile and body composition, which reinforces the need to establish specific criteria in terms of anthropometric predictors, since the body characteristics of women from Alagoas are different from those used in the samples investigated by international organizations and that formed the basis to the establishment of the criteria defined by such agencies.^[6,7,10]^

The short stature is an independent risk factor for the development of obesity and hypertension.^[21,22]^ There is an argument that states that the inverse association between stature and obesity or hypertension is given because the height is a strong marker of the nutritional status in early stages of life. Inadequate nutrition in these critical stages, especially in the intrauterine and immediate postnatal phases, induces persistent changes in cell number, structure of organs, physiology, and body metabolism. These adaptive mechanisms, known as metabolic imprinting, induces the individual to become more susceptible to obesity, hypertension, and other chronic diseases in adulthood.^[23,24]^

The prevalence of hypertension in women from Alagoas (21.8%) was similar to that value estimated for all the country (24.2%) and for the Northeastern region (19.4%), at the time of on the occasion of the National Survey of Health, in 2013.^[2]^

Obesity was present in 28.2% of the assessed population, a higher value than that one found in Brazilian women (16.9%) according to the Family Expenditure Survey (2008/2009).^[25]^ In these women, the prevalence of hypertension was 2.5 times higher than in those women with BMI <30 kg/m^2^. Furthermore, comparing the prevalence of hypertension among women with “general” obesity (BMI ≥30 kg/m^2^) with those women with abdominal obesity (WC >80 cm), it was observed that those women, with fat accumulation in the central region of the body, presented prevalence 30% higher. This relationship shows the importance of identifying the pattern of the body fat distribution through anthropometric techniques.

Despite presenting multifactorial genesis, not fully clarified, some mechanisms are seen as mediators of obesity influence on the hypertension development. Among them, we can mention the increased insulin secretion, the inadequate activation of the renin–angiotensin–aldosterone system, an increased activity of the sympathetic nervous system, an increased oxidative stress, as well as the action of adipokines.^[26]^

Confirming this relationship, the correlation analysis between the anthropometric indicators and BP variables showed that, in all situations, the association was positive and statistically significant. In other words, as the body proportions increased, the pressure levels followed the same trend.

The correlation coefficients, although statistically significant, suggest a weak association. However, it is important to highlight that the use of anthropometric indicators has the purpose of screening individuals at increased risk, and not to establish the hypertension diagnosis. Therefore, this strength of association is shown consistent with the expected situation, and agrees with previous studies that involved anthropometric predictors of hypertension conducted in Brazil and in other countries.^[27–30]^

All the assessed anthropometric parameters showed reasonable accuracy as a screening method for high BP. Additionally, when we compare the areas under the ROC curves, WHtR was the predictor with the best performance. In this way, the data confirm that, for the female population from Alagoas, aged 20 to 49, the WHtR indicator is the one with greater discriminatory power, in terms of hypertensive risk screening.

This is the first study to highlight the statistical superiority of WHtR as a hypertension screening method in Brazilian women (especially if the 0.54 cut-off point is used). Similar results were found in studies carried out in the Asian female population,^[29–33]^ whose racial and physical characteristics are noticeably different from the Western female population.

The association between abdominal obesity and hypertension indicates that this pattern of body fat distribution can maximize the hemodynamic changes caused by obesity. The adipocytes, concentrated in the visceral area, have a more biologically active metabolism, tending to release greater amounts of fatty acids and proinflammatory adipokines. Thus, the insulin resistance aspect gets worse, as well as the endothelial dysfunction, dysregulation of hepatic metabolism and, in consequence, it favors the onset of cardiovascular disorders.^[5]^

However, even the abdominal obesity indicators showed differentiated discriminatory power. It is believed that the greater accuracy of WHtR is due to the incorporation of height values in its calculation,^[34]^ because this variable shows hypertension and obesity association, as already mentioned^[23,24]^ in this study. Therefore, this result may have been influenced by the lower average stature of the population from Alagoas, in comparison with the other population groups.

Additionally to choosing the most appropriate anthropometric predictor, it is essential to determine ideal cut-offs points, in order to optimize the accuracy of screening tests and, therefore, reduce the proportion of inconsistent results.^[7]^ In screening tests, the results that are wrongly marked as positive, can favor the increasing of demands in the health service, burdening professionals and raising public expenditures. In contrast, when individuals at real risk are not identified, the false-negative tests contribute to the extension of the deleterious effects on health.^[35]^

The ideal cut-off points of the anthropometric parameters for women from Alagoas were significantly associated with high BP, even after the control of possible confounding factors, such as age, race, economic class, education, salt intake, and level of physical activity. Therefore, we can confirm the accuracy of these tools as a screening method for hypertension risk.

Regarding the WHtR, through a meta-analysis of articles published until mid-2008, Browning et al^[36]^ suggest the standardization of the cut-off point of 0.50 for hypertension identification, regardless of age or sex, so diverging from the value of 0.54, identified in this study as an ideal value. However, 63% of the studies included in this review involved the Asian population. In Brazil, a study carried out in Vitória (ES) found that the 0.53 value, applied to the WHtR, was the best cut-off point for women in hypertension screening.^[27]^

The WC has emerged as the second best anthropometric indicator for the screening of high BP. This anthropometric indicator is that one the most recommended by international organizations, for metabolic risk identification. In general, 2 cut-off points are recommended: >80 and >88 cm.^[6,10,37]^ The risk of the problem would be overestimate, in case of taking the first one as a parameter for hypertension screening in women from Alagoas. The second cut-off point determines results similar to that one more accurate, considering the population now studied (89 cm). Among Brazilian studies with the same purpose, the best results were obtained with lower cut-offs points, ranging close to 80 cm.^[27,38–40]^ In the international context, we can highlight the wide range of identified cut-off point values as appropriate for hypertension prediction in women (65–95 cm).^[36]^

The WC is an anthropometric indicator of abdominal obesity simple and easy to obtain and interpret. However, its limitation refers to the low precision in assuming the same cut-off point for individuals with different statures, since they also present unequal body proportions.^[41]^ Therefore, corroborating the data obtained in this article, Ashwell et al,^[34]^ through meta-analysis results, showed the superiority of the WHtR, in comparison with the other anthropometric parameters, as a screening tool for high BP.

Also in relation to abdominal fat indicators, an identical value, to the one found for the female population from Alagoas, was appointed to WHR in the MONICA-WHO study (0.83).^[27]^ Although being a lower ratio, it is close to the cut-off point recommended by the WHO for identifying increased metabolic risk in women (>0.85).^[6]^ Despite the effectiveness of the WHR as an indicator of body fat distribution pattern, some authors reject its use in the monitoring context, since both waist and hip can decrease or increase, depending on the weight change. Thus, the ratio itself is minimally altered.^[36]^

Among all assessed anthropometric indicators, the C index was the parameter that showed the lower hypertension discriminatory power, supporting a population-based study carried out in the city of Florianópolis.^[39]^ In this study, Silva et al detected a value of 1.15 as the optimal value of C index, to detect the hypertension risk in predominantly Caucasian women (86%). An investigation in a population with close characteristics to the population of Alagoas, carried out in the city of Salvador (BA), detected the 1.18 value as the best cut-off point to identify high coronary risk, a quite similar value than the one shown in this study (1.19).^[42]^ However, the authors did not evaluate the hypertension risk in an isolated way.

The best cut-off point for hypertension screening, using BMI in this population (29.1 kg/m^2^) is established within the overweight diagnostic limits (25.0–29.9 kg/m^2^),^[7]^ as seen in most studies involving non-Asian female populations. In Asian women, the values detected fluctuated below this limit,^[43]^ an condition that contributed to arise discussion about the need for an BMI reclassification for this population.^[44]^ Despite its inability to differentiate body compartments or proportions,^[45]^ due to its direct relation with the general fat concentration, we cannot deny the BMI importance as an educational and monitoring parameter for the general population, as well as its usefulness to epidemiological studies.

In this study, the best %BF cut-off point for high BP prediction was considerably lower than that one estimated for Mexican women (33% vs 44%).^[46]^ However, this comparison becomes affected because of methodological differences, both in the %BF measurement (DEXA) as well as in the cut-off point selection. On the other hand, the findings disclosed in this study approached to those found in studies carried out in Brazil (34%) and Bangladesh (32%), that estimated the %BF using predictive equations, involving age and anthropometric measurements.^[47,48]^

It is possible that the observed variations between studies should result from the distinctions existing in the body composition between populations, even within the same country,^[49]^ especially in those countries with continental dimensions, such as Brazil. This aspect reinforces the need to assess the validity of the predictors, in each context. Additionally, other factors can affect the accuracy of a diagnostic test, the prevalence or severity of the disease, the study design and the sample selection.^[50]^ The differences between cut-off points can also be explained by different methodologies used in their determination.

The choice of an anthropometric predictor in a metabolic risk screening involves other factors besides the test accuracy, such as the equipment availability and skilled professionals. Because of these possible limitations, this study assessed the best cut-off points for several anthropometric parameters, and not only for that one that showed a greater discriminatory power. Thus, we can extend the ability to use these parameters in the hypertension screening.

A limitation of this investigation was the fact that the 2 BP measurements were taken almost simultaneously. The criteria for clinical diagnosis of hypertension establishes that at least 2 readings are performed at different moments.^[3]^ Otherwise, there may be an overestimation of the prevalence of hypertension. As a consequence, higher sensitivity is obtained (in detriment of the specificity). This is a common problem to the screening tools, because high sensitivity and specificity are characteristics of procedures for diagnostic confirmation (gold standard).^[51]^ In summary, for screening it is better to have false positives than false negatives.

We can conclude that WHtR indicator, with a 0.54 cut-off point value, is the most accurate resource for screening women from Alagoas, at risk of high BP. People thus identified should be referred for clinical confirmation of diagnosis to, if necessary, be inserted into education programs in health, and be oriented on the need to adopt healthy lifestyle habits and, of course, receive the appropriate treatment for hypertension. Thus, anthropometric predictors may help to optimize the hypertension detection and the establishment of appropriate measures of prevention and control.

Because of the relevance of the subject, we suggest to examine the predictive capacity of anthropometric indicators in male and child populations in the state of Alagoas, as well as in other national and international scenarios.

In the absence of specific studies and considering the largest ethnic proximity and the environmental and epidemiological similarity, the findings now obtained can be extended to women of other Brazilian states, especially those in the Northeastern region.

## Acknowledgments

The author would like to thank the Instituto Brasileiro de Geografia e Estatística (IBGE), for the assistance in identifying the census sectors. And to the City Halls of the visited municipalities, to the community health workers, and to the population studied for the local support to researchers. To all the researchers involved in the study, for their assistance in collecting and tabulating data.
